# *Heilipussquamosus* (LeConte, 1824): clarification of the correct name for the “avocado tree girdler” with updates on its biology and distribution (Coleoptera, Curculionidae, Molytinae, Hylobiini)

**DOI:** 10.3897/BDJ.10.e85499

**Published:** 2022-09-30

**Authors:** Salvatore S Anzaldo, Valentina Díaz-Grisales

**Affiliations:** 1 Arizona State University, Tempe, United States of America Arizona State University Tempe United States of America; 2 Programa de Entomología y Acarología, Colegio de Postgraduados, Campus Montecillo, Montecillo, Mexico Programa de Entomología y Acarología, Colegio de Postgraduados, Campus Montecillo Montecillo Mexico

**Keywords:** weevil, Lauraceae, host associations, new state record, *Persea*

## Abstract

**Background:**

A single species of the genus *Heilipus* Germar, 1824 is native to the south-eastern United States and was a pest of avocado in Florida in the mid-20^th^ century. Two names—*Heilipusapiatus* (Olivier, 1807) and *H.squamosus* (LeConte, 1824)—have both recently been used as the valid name for this species, with *H.apiatus* also being recently used as the valid name for a species from French Guiana. Confusion surrounding the correct application of these names and the distribution of these species traces back to an erroneous distribution given in Olivier's 1807 description of *H.apiatus* and, although some authors clarified this previously, there continues to be confusion concerning the correct name. Outside of avocado-producing areas, this species was rarely collected and little was known about its biology. Recent observations on iNaturalist and BugGuide suggest the species is more widely distributed in the United States and less rare than it was previously thought to be.

**New information:**

*Heilipussquamosus* (LeConte, 1824) is recognised as the valid name for the sole species of *Heilipus* occurring in the United States, while *Heilipusapiatus* (Olivier, 1807) is a very different species from French Guiana. *Heilipussquamosus* was previously recorded from eight States in the south-eastern United States and, after examining records from natural history collections, iNaturalist, BugGuide and literature sources, the species is newly recorded from an additional seven States: Arkansas, Kansas, Kentucky, Louisiana, Missouri, Oklahoma and Texas. Though native host plants have been unconfirmed by rearing records, the evidence indicating the possible host plants in the plant family Lauraceae is reviewed.

## Introduction

The genus *Heilipus* Germar, 1824 (Germar 1824) contains 88 species in the New World (O'Brien and Wibmer 1982, Wibmer and O'Brien 1986, Díaz et al. 2021), with a single species occurring in the United States (O'Brien and Wibmer 1982, Anderson 2002). The species described as *Rhynchaenusapiatus* Olivier, 1807 and *Pissodessquamosus* LeConte, 1824 were both originally indicated to be from the south-eastern United States (Olivier 1807, LeConte 1824). The introduction of South American countries to the published distribution of *H.apiatus* (Schoenherr 1836, Berg 1881) added confusion to the identity of this species which persists today. The two species were synonymised by Blatchley and Leng (1916) in the genus *Heilipus*, with Olivier's name (*H.apiatus*) having precedence. Since this act, the valid name for the species of *Heilipus* occurring in the United States has been inconsistently used, with *H.squamosus* (LeConte) and *H.apiatus* (Olivier) both being used in the 20^th^ and 21^st^ centuries.

On three occasions, the confusion surrounding the distribution and valid names for these species appeared to have been resolved, but was overlooked by subsequent authors. First, Schoenherr (1836) includes both Cayenna and Florida in the distribution for *H.apiatus*, but only Cayenna is listed in a later volume (Schoenherr 1843). This possibly indicates awareness of an erroneous North American distribution originally given by Olivier, but no clarification was given. Later authors (e.g. Gemminger and Harold 1871, Berg 1881) include both North and South America in the distribution of *H.apiatus* again. Second, Wolfenbarger (1950) references the synonymy of Blatchley and Leng (1916) and states, “This synonymity is accepted as an error since Dr. [William H.] Anderson reported that, ‘Olivier’s species is quite different and occurs in South America'”. *Heilipusapiatus* continued to be used by Woodruff (1963) and O'Brien and Wibmer (1982) for the U.S. species without reference to Wolfenbarger’s statement (Wolfenbarger 1950). Third, O'Brien and Wibmer (1984) correct their previous use of *H.apiatus* (*O'Brien and Wibmer 1982*) in the often overlooked supplement to their checklist, stating that the type locality of *H.apiatus* as Florida is an error and that it is a South American species, making *H.squamosus* (LeConte) the valid name for the species from the United States. In their later treatment on South American weevils, Wibmer and O'Brien (1986) accordingly list *H.apiatus* (Olivier) as a species from French Guiana.

**Figure 1.**
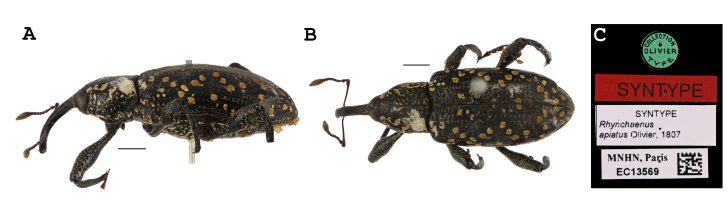
*Heilipusapiatus* syntype (MNHN EC13569). Scale bars = 2 mm. Photographs copyright Maxime Le Cesne/MNHN. A: lateral habitus. B: dorsal habitus. C: labels.

Since the latest clarification by O'Brien and Wibmer (1984), however, the name *H.apiatus* has persisted as the valid name for the species in the United States in published literature and online resources. The name *H.apiatus* has also been used correctly recently, following Wibmer and O'Brien (1986), for the species from French Guiana (Gombauld 2004, Rheinheimer 2010, Rheinheimer 2017). Examination of images of Olivier’s *H.apiatus* syntypes (Fig. 1) reconfirms the positions taken by Schoenherr (1836), Wolfenbarger (1950) and O'Brien and Wibmer (1984)—*H.apiatus* and *H.squamosus* are distinct species and *H.apiatus* is not the species found in the United States.

## Materials and methods

One hundred and thirty-five occurrence records of *H.squamosus* were analysed (Suppl. material 1). Fifty-seven of those records are preserved specimens, only 10 of which are digitised records available online (SCAN 2022, Ecdysis 2022, GBIF.org 2022b) and are housed in the following natural history collections:

- **ABS** – Archbold Biological Station Arthropod Collection, Lake Placid, FL
- **ASUCOB** – Arizona State University Charles W. O’Brien Collection, Tempe, AZ
- **AUMNH** – Auburn University Museum of Natural History, Auburn, AL

The remaining 47 records of preserved specimens were not previously available online; information for these records is available in Suppl. material 1 and GBIF.org (2022a). The following collections provided specimen data and/or photographs of their *H.squamosus* material:

- **CMNC** – Canadian Museum of Nature Collection, Ottawa, Canada
- **FSCA** – Florida State Collection of Arthropods, Gainesville, FL
- **LSAM** – Louisiana State Arthropod Museum, Baton Rouge, LA
- **MEM** – Mississippi Entomological Museum, Mississippi State, MS
- **NCSU** – North Carolina State University Insect Collection, Raleigh, NC
- **UGCA** – University of Georgia Collection of Arthropods, Athens, GA
- **VMNH** – Virginia Museum of Natural History, Martinsville, VA

In addition to specimens from natural history collections, 43 observations posted on the websites BugGuide (VanDyk 2021) and iNaturalist (iNaturalist 2022) that could be verified as being *H.squamosus* (all identified as *H.apiatus*) were also analysed (Suppl. materials 1, 2). Of these records, 21 are published to GBIF, including 14 research-grade iNaturalist observations (GBIF.org 2022b).

Literature sources citing *H.squamosus* occurrences from additional localities (Blatchley and Leng 1916, Woodruff 1963, Hoffman 2003, Ciegler 2010, Fraedrich et al. 2011) were also included. Records of any type without at least county-level locality data (fewer than 10 overall) were excluded from further analysis.

**Figure 2.**
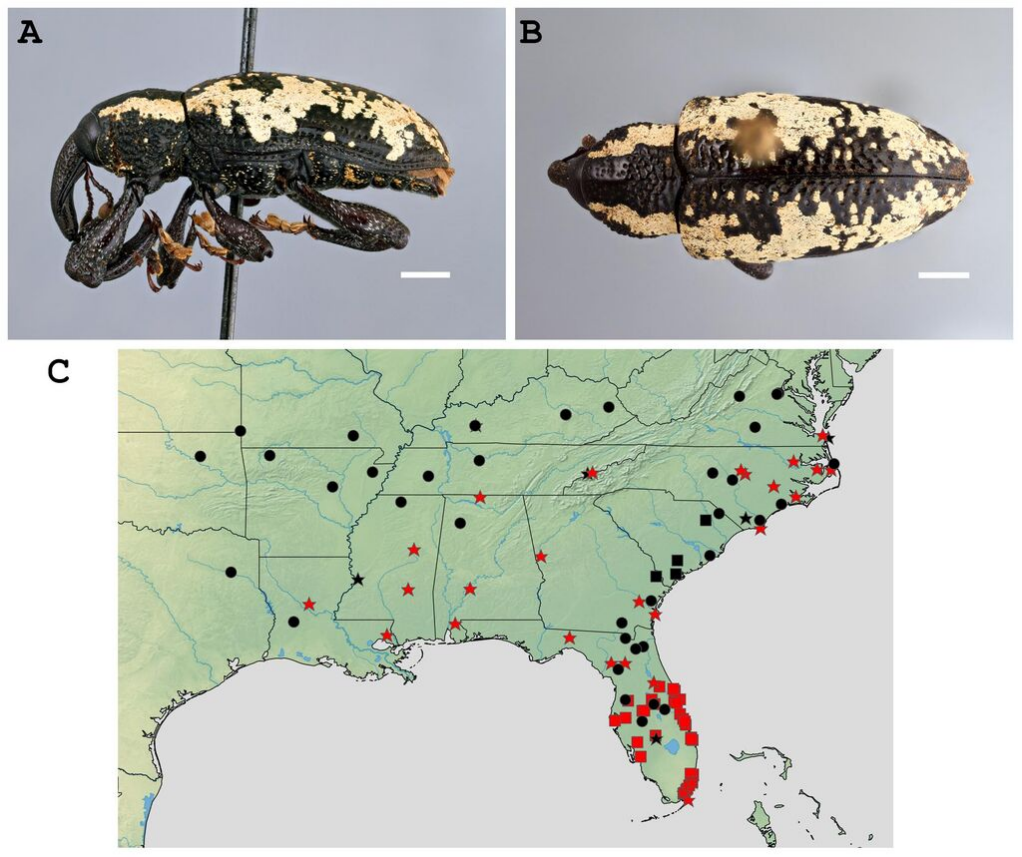
*Heilipussquamosus*. Scale bars = 2 mm. A: lateral habitus. B: dorsal habitus. C: distribution map (circles = iNaturalist/BugGuide records, stars = specimen data, squares = literature records; red symbols = 20^th^ century records, black symbols = 21^st^ century records).

Botanical names used are the accepted name from Tropicos.org (2021) at the time of access. Photographs of the syntype series of *H.apiatus* were provided by the Muséum National d’Histoire Naturelle (MNHN; Fig. 1). Habitus images of authoritatively identified *H.squamosus* (Fig. 2A-B) were taken with a BK Lab imaging system with a Canon EOS 7D camera equipped with a 65 mm macro lens. Images were stacked with Zerene Stacker. Distribution maps were created with SimpleMappr (Shorthouse 2010; Fig. 2C).

## Taxon treatments

### Heilipus squamosus

(LeConte, 1824)

75391CCE-AEDD-5085-93DB-900086002078

**Original combination**: *Pissodessquamosus* LeConte, 1824: 161
Description: https://www.biodiversitylibrary.org/page/15913340
Holotype: https://mczbase.mcz.harvard.edu/guid/MCZ:Ent:5176
**Synonyms**:
Heilipus squamosus Boheman, 1836: 171 (not LeConte, 1824).
Description: https://www.biodiversitylibrary.org/page/4109434

#### Distribution

The previously-published state distribution of *Heilipussquamosus* is in the south-eastern United States—Georgia (LeConte 1824), Florida (LeConte and Horn 1876), Tennessee (Blatchley and Leng 1916), South Carolina, Alabama and Mississippi (Wolfenbarger 1950), North Carolina (O'Brien and Wibmer 1982) and Virginia (Hoffman 2003). The species was considered rare (LeConte 1824, LeConte and Horn 1876, Barber 1912) and, apart from specimens from Florida where the species was a pest on avocado, relatively few specimens of this large and distinctive weevil have been collected and deposited in natural history collections.

In the past 20 years, citizen science initiatives (e.g. BugGuide, iNaturalist) have yielded observations of *H.squamosus* from all States in its previously reported range plus seven additional States, expanding the distribution of this species to the northern and western regions of the United States:

Alabama, Arkansas (**new State record**), Florida, Georgia, Kansas (**new State record**), Kentucky (**new State record**), Louisiana (**new State record**), Missouri (**new State record**), Mississippi, North Carolina, Oklahoma (**new State record**), South Carolina, Tennessee, Texas (**new State record**) and Virginia (Fig. 2C).

#### Biology

The biology of *H.squamosus* is still incompletely known. Before it became a pest of non-native avocado in Florida, the published natural history information was limited to a record from “under pine bark” (LeConte and Horn 1876: 141) and from “budding shoots of sassafras” (Blatchley and Leng 1916: 187). The avocado industry in Florida traces back to the early 20^th^ century, though *Perseaamericana* Mill. was first brought to and became naturalised in Florida before 1835 (Wolfe et al. 1942). Wolfenbarger (1948) reports the earliest evidence of *H.squamosus* causing damage to avocado to be from the late 1930s and, by the late 1940s, the weevil became “one of the most dangerous pests of the avocado” (Wolfe et al. 1949). Larvae feed under the bark near ground level and can girdle trees and cause an 8–10% loss of avocado trees in a grove (Wolfenbarger 1948). The status of *H.squamosus* as a pest appears to have continued through the 1950s, but the species was rarely observed after (Wolfenbarger 1971).

The native hosts for the weevil have been suspected to be other members of the Lauraceae, namely *Linderamelissifolia* (Walter) Blume (pondberry), *Litseaaestivalis* (L.) Fernald (pondspice), *Perseaborbonia* (L.) Spreng. (redbay) and *Sassafrasalbidum* (Nutt.) Nees (sassafras), though no definitive rearing records are known. Of these species, sassafras is widely distributed throughout the eastern United States; the other three are restricted to coastal plains and swampy areas, with pondberry and pondspice being listed as a federally endangered and threatened species, respectively.

Adult weevils have been found on pondberry in North Carolina (U.S. Fish and Wildlife Service 1993) and on pondspice in Georgia (Cameron et al. 2008, Fraedrich et al. 2011). An unidentified weevil larva described as being a similar size to the adult *H.squamosus* was found causing feeding damage to the root collar at the base of a pondberry plant in South Carolina (Fraedrich et al. 2011). Redbay was suggested as a possible host due to the weevil’s distribution along the East Coast (Barber 1912, Hoffman 2003); Cameron et al. (2008) observed debarking damage to redbay branches which was noted to be very similar to the damage observed on pondspice branches at a locality in Georgia where *H.squamosus* was also found, but no more direct associations are known with redbay. Adult weevils have been collected on sassafras in Tennessee (Blatchley and Leng 1916) and Alabama [AUMNH].

Additional records: Wolfenbarger (1948) reported specimens collected “on cotton, sassafras and Satsuma orange” and, in a subsequent paper (Wolfenbarger 1950), he adds “camphor” to the list. Camphor, *Cinnamomumcamphora* (L.) J. Presl, is a non-native species of Lauraceae naturalised in parts of south-eastern United States. No additional records were found indicating an association of *H.squamosus*. Three specimens have been collected from Isle of Wight Co., Virginia on *Diospyrosvirginiana* L. (wild persimmon; Ebenaceae) (CMNC; Art Evans, *pers. comm*.). One specimen from Spotsylvania Co., Virginia was photographed “on dogbane hemp”, *Apocynumcannabinum* L. (Apocynaceae) (iNaturalist, BugGuide).

## Discussion

*Heilipussquamosus* was previously known from eight States in the south-eastern United States and is herein reported from an additional seven States, expanding the distribution in the country to the west and north. The reason for the poorly-documented distribution is likely related to the weevil’s rarity, as well as a possible recent range expansion. The species has been historically reported to be rare and this is supported by only 57 adult specimens of this large and distinctive species being located in natural history collections. Removing records from Florida, where *H.squamosus* is known to be a pest on avocado, 33 specimens were found from only nine of the other 14 States where the species is now recorded. The rarity of the species can possibly be attributed to the rarity of the potential host plants: pondberry and pondspice. Pondberry is thought to have been uncommon even before recent habitat destruction and extant populations are known from only a few counties in each State in its range (Devall 2013). Of the newly-recorded States, two of three records from Arkansas and the only record from Missouri occur either in the same county (Craighead Co., AR) or a neighbouring county (White Co., AR; Carter Co., MO) to where the few extant populations of pondberry are known in these States (Devall 2013). The distribution of pondberry and pondspice alone do not account for the full distribution of *H.squamosus* as both plant species are absent from Kansas, Kentucky, Oklahoma, Tennessee and Texas (Gramling 2010, Devall 2013). Redbay ranges into east Texas and, while sassafras is present in each State, it apparently does not occur as far east as Tulsa Co., OK, where *H.squamosus* was observed (Randolph 2017). Other native Lauraceae, such as *Linderabenzoin* (L.) Blume, do occur in the full range of *H.squamosus*, but no associations of the weevil with these plants are known.

Another explanation for the poorly-documented distribution is that the range of *H.squamosus* has recently expanded. In the 21^st^ century, citizen science initiatives like BugGuide and iNaturalist have provided an easy way for users to contribute observation data. Forty-three additional records of *H.squamosus* came from these sources from all 15 States from where the weevil is currently recognised, including the only known observations from five of the seven newly-recorded States (Fig. 2C, black circles). Such a large and distinctive species would be expected to be present in natural history collections if it had occurred in a region for a long time. So far, no specimens collected in Arkansas, Kansas, Missouri, Oklahoma or Texas were found in collections, suggesting that the weevil has not been present in these States for very long. All known records for those five States plus Kentucky have occurred in the 21^st^ century (Fig. 2C, black circles). The iNaturalist observation data also indicate that *H.squamosus* may not actually be rare in the newly-recorded States. Seven total specimens were observed from Tulsa County, Oklahoma and Madison County, Kentucky since July 2020—the same number of records that have been found from all sources from the State of Georgia, from which *H.squamosus* has been recorded for nearly two centuries!

Native Lauraceae, including the possible host plants discussed here, are threatened by the spread of laurel wilt disease (Cameron et al. 2008, Gramling 2010, Fraedrich et al. 2011, Devall 2013, Randolph 2017, Best and Fraedrich 2018). It is currently unclear whether this, or other factors such as climate change, are driving changes to the distribution and/or abundance of the weevil historically known only in the southeast. While the only avocado-producing region in which *H.squamosus* is currently known to occur is Florida, the ability of this species to live in 14 other States indicates that the species might also be able to continue spreading to and thrive in other avocado-producing regions (e.g. California). Locating more natural history collection specimens and making new observations will reveal more about the biology and distribution of this incompletely known species.

### Conclusion

- *Heilipussquamosus* (LeConte, 1824) is the valid name for the single species of *Heilipus* occurring in the United States. Much of the prior literature and current internet resources refer to this species as *H.apiatus* (Olivier, 1807), which is an incorrect name for this species.
- *Heilipussquamosus* was previously recorded from Alabama, Florida, Mississippi, North Carolina, South Carolina, Tennessee and Virginia and is newly recorded from Arkansas, Kansas, Kentucky, Louisiana, Missouri, Oklahoma and Texas.
- Possible host plants of *H.squamosus* include *Linderamelissifolia* (Walter) Blume (pondberry), *Litseaaestivalis* (L.) Fernald (pondspice), *Perseaborbonia* (L.) Spreng. (redbay) and *Sassafrasalbidum* (Nutt.) Nees (sassafras), which are all species of Lauraceae, although larval associations with these plants are currently limited.
- *Heilipusapiatus* (Olivier, 1807) is a valid name that applies to a species known only from French Guiana.

## Supplementary Material

XML Treatment for Heilipus squamosus

C82B0DE4-DE7C-5EEC-A782-1DE0563F9FAD 10.3897/BDJ.10.e85499.suppl1 Supplementary material 1 *Heilipussquamosus* records observed in this study, separated by record type Data type Excel spreadsheet Brief description An Excel spreadsheet with three tables: 1) iNaturalist and BugGuide observations, 2) natural history collection specimens and 3) literature records. File: oo_733770.xlsx https://binary.pensoft.net/file/733770 Salvatore Anzaldo. Data provenance: cited natural history collections, repositories and authors.

9C9F67E2-3758-5A60-A3AF-312C442FA4B4 10.3897/BDJ.10.e85499.suppl2 Supplementary material 2 iNaturalist records of *H.squamosus* Data type Observation data in a .csv file. Brief description The file includes all records (not only research grade) on iNaturalist at the time of access pertaining to the species *Heilipussquamosus*. File: oo_630717.csv https://binary.pensoft.net/file/630717 iNaturalist
